# Platelet Glycoprotein Ib α‐Chain as a Putative Therapeutic Target for Juvenile Idiopathic Arthritis: A Mendelian Randomization Study

**DOI:** 10.1002/art.41561

**Published:** 2021-02-21

**Authors:** Shan Luo, Sarah L. N. Clarke, Athimalaipet V. Ramanan, Susan D. Thompson, Carl D. Langefeld, Miranda C. Marion, Alexei A. Grom, C. Mary Schooling, Tom R. Gaunt, Shiu Lun Au Yeung, Jie Zheng

**Affiliations:** ^1^ The University of Hong Kong, Hong Kong, China, and University of Bristol Bristol UK; ^2^ University of Bristol and University Hospitals Bristol NHS Foundation Trust Bristol UK; ^3^ University of Cincinnati College of Medicine and Cincinnati Children’s Hospital Medical Centre Cincinnati Ohio; ^4^ Wake Forest School of Medicine Winston‐Salem North Carolina; ^5^ Cincinnati Children’s Hospital Medical Centre Cincinnati Ohio; ^6^ The University of Hong Kong, Hong Kong, China, and The City University of New York School of Public Health and Health Policy New York; ^7^ University of Bristol and NIHR Bristol Biomedical Research Centre Bristol UK; ^8^ The University of Hong Kong Hong Kong China; ^9^ University of Bristol Bristol UK

## Abstract

**Objective:**

To ascertain the role of platelet glycoprotein Ib α‐chain (GPIbα) plasma protein levels in cardiovascular, autoimmune, and autoinflammatory diseases and whether its effects are mediated by platelet count.

**Methods:**

We performed a two‐sample Mendelian randomization (MR) study, using both a *cis*‐acting protein quantitative trait locus (*cis*‐pQTL) and *trans*‐pQTL near the *GP1BA* and *BRAP* genes as instruments. To assess if platelet count mediated the effect, we then performed a two‐step MR study. Putative associations (GPIbα/platelet count/disease) detected by MR analyses were subsequently assessed using multiple‐trait colocalization analyses.

**Results:**

After correction for multiple testing (Bonferroni‐corrected threshold *P* ≤ 2 × 10^−3^), GPIbα, instrumented by either *cis*‐pQTL or *trans*‐pQTL, was causally implicated with an increased risk of oligoarticular and rheumatoid factor (RF)–negative polyarticular juvenile idiopathic arthritis (JIA). These effects of GPIbα appeared to be mediated by platelet count and were supported by strong evidence of colocalization (probability of all 3 traits sharing a common causal variant ≥0.80). GPIbα instrumented by *cis*‐pQTL did not appear to affect cardiovascular risk, although the GPIbα *trans*‐pQTL was associated with an increased risk of cardiovascular diseases and autoimmune diseases but a decreased risk of autoinflammatory diseases, suggesting that this *trans‐*acting instrument operates through other pathways.

**Conclusion:**

The role of platelets in thrombosis is well‐established; however, our findings provide some novel genetic evidence that platelets may be causally implicated in the development of oligoarticular and RF‐negative polyarticular JIA, and indicate that GPIbα may serve as a putative therapeutic target for these JIA subtypes.

## INTRODUCTION

Platelet glycoprotein Ib α‐chain (GPIbα) is a platelet surface membrane protein (1). It functions as a receptor for von Willebrand factor (vWF) and is implicated in atherothrombosis (2). Genetic evidence supports the assertion that GPIbα influences atherothrombosis via increased platelet counts (3). Given the potential of GPIbα as an antithrombotic target, its efficacy for the treatment of thrombotic thrombocytopenic purpura is currently being investigated in a phase II trial (3). Recent studies have also indicated a role for platelets in inflammation and immunity (4, 5), which may imply potential for repurposing GPIbα as a target for prevention/treatment of immune‐related disease. However, these putative associations have not been systematically evaluated.

Mendelian randomization (MR) studies utilize genetic variants, randomly allocated during conception, as instruments to infer causality and are less prone to confounding and reverse causation than observational studies (6). They are increasingly used to ascertain the health effects of potential therapeutic targets. Colocalization can further help to distinguish causal effects from confounding via linkage disequilibrium (LD) (7). Collectively, applying MR and colocalization to ‐omics data can provide a distinct strand of genetic validation for putative causal gene targets and thus improve the success rate of drug trials (8, 9).

To understand the effects of GPIbα on cardiovascular, autoimmune, and autoinflammatory diseases, and whether these are mediated by platelet count, we conducted a two‐step, two‐sample MR study. We subsequently performed multiple‐trait colocalization analyses (i.e., on GPIbα, platelet count, and a disease) to complement the evidence for causal associations detected in our MR study.

## MATERIALS AND METHODS

#### Study design

MR relies on 3 core assumptions. First, the genetic variant is robustly associated with the exposure. Second, the genetic variant is independent of confounders of the exposure–outcome association. Third, the genetic variant is independent of the outcome except via the exposure (10).

**Figure 1:**
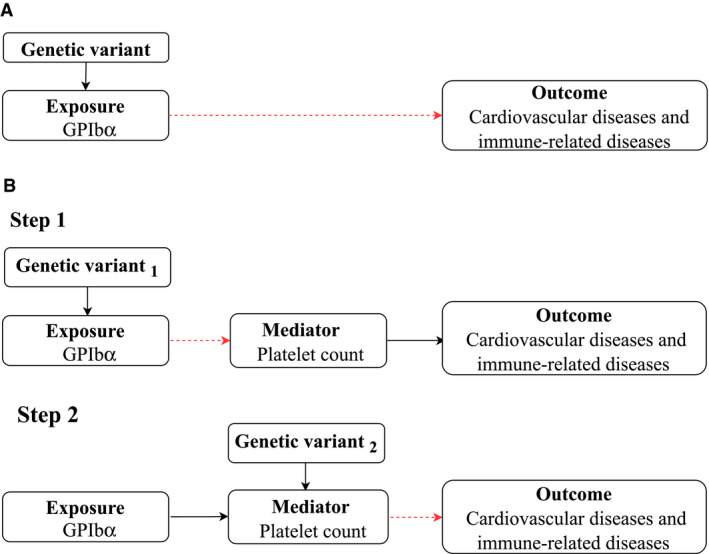
Schematic diagram of **A**, standard Mendelian randomization (MR) analysis of glycoprotein Ib α‐chain (GPIbα) and **B**, two‐step MR analysis of mediation by platelet count. Two‐step MR tests the association between a genetic variant and the exposure (GPIbα) postulated to influence the outcome (cardiovascular and immune‐related diseases) via an altered mediator (platelet count). Broken arrows indicate the causal pathway to be assessed. Color figure can be viewed in the online issue, which is available at http://onlinelibrary.wiley.com/doi/10.1002/art.41561/abstract.

In this study, we first performed a two‐sample MR study to assess the association of GPIbα with cardiovascular, autoimmune, and autoinflammatory disease risk (Figure 1A). To further assess whether platelets mediate the effects of GPIbα on disease, we subsequently performed a two‐step, two‐sample MR study. First, we assessed the association of GPIbα with platelet count. Second, we assessed the effect of platelet count on the disease outcome (Figure 1B).

#### Genetic instruments of GPIbα

A proteome genome‐wide association study (GWAS) was conducted in 3,301 healthy blood donors of European ancestry (3) randomly selected from the INTERVAL study (50% male) (11). The plasma protein concentrations were quantified by aptamer‐based multiplex protein assay (SOMAscan) (3). Genotyping was performed on an Affymetrix Axiom array and was imputed using a combined 1000 Genomes Phase 3‐UK10K reference panel (3). Genetic variants were excluded if they had a call rate of <99%, had a minor allele count of <8, deviated from Hardy‐Weinberg equilibrium (*P* < 5 × 10^−6^), or had an info score of <0.7 (3). The genetic associations were obtained in an additive genetic model adjusted for age, sex, duration between blood draw and processing, and the first 3 principal components of ancestry (3). Conditionally uncorrelated variants (with the lowest *P* value having LD r^2^ < 0.001) associated with GPIbα (*P* < 5 × 10^−8^) were selected as instruments.

#### Genetic instruments of platelet count

At the time of analyses, the largest hematologic GWAS that had been conducted included 173,480 participants of European ancestry (12). Participants were from the UK Biobank (n = 132,959; 48% male) and the INTERVAL studies (n = 40,521; 50% male) (11). Complete blood cell count was performed using a combination of fluorescence and impedance flow cytometry within 36 hours (12). Genotyping was undertaken using Affymetrix UK BiLEVE and UK Biobank Axiom arrays, and imputation was to a reference set combining the UK10K and Haplotype Reference Consortium reference panels (12). Genetic associations were obtained from a linear mixed model adjusted for the top 10 principal components of ancestry and recruitment center (12). Conditionally uncorrelated variants (with the lowest *P* value having LD r^2^ < 0.001) associated with platelet count (*P* < 8.31 × 10^–9^, a threshold for common, low frequency, and rare variants) (13) were selected as instruments. Since the genetic instruments for GPIbα were also strongly associated with platelet count, we undertook a sensitivity analysis which estimated the instrument‐specific effect of platelet count on the diseases of interest.

#### Genetic associations of selected outcomes

Outcomes included platelet count (per nl), 10 major cardiovascular diseases (coronary heart disease [CHD], myocardial infarction [MI], arterial embolism and thrombosis, deep venous thrombosis [DVT], phlebitis and thrombophlebitis, any stroke, any ischemic stroke, cardioembolic stroke, large artery stroke, and small vessel stroke), and 12 immune‐related diseases. Immune‐related diseases were classified (14) as autoimmune diseases (type 1 diabetes mellitus [type 1 DM], juvenile idiopathic arthritis [JIA; oligoarticular and rheumatoid factor [RF]–negative polyarticular subtypes], rheumatoid arthritis [RA], systemic lupus erythematosus, psoriasis, multiple sclerosis, primary sclerosing cholangitis, and primary biliary cirrhosis), autoinflammatory diseases (inflammatory bowel disease [IBD], Crohn's disease [CD], and ulcerative colitis [UC]), or atopic disease (eczema) (15). We obtained summary genetic associations (including estimates of regression coefficient, the corresponding standard error and *P* value, effect allele, other allele, and effect allele frequency) for each outcome from the largest publicly available GWAS at the time of analyses (16, 17, 18, 19, 20, 21, 22, 23, 24, 25, 26, 27) (Supplementary Table 1, available on the *Arthritis & Rheumatology* website at http://onlinelibrary.wiley.com/doi/10.1002/art.41561/abstract).

#### MR analysis

To estimate the effect of exposure on outcome (β_XY_), we used the Wald estimate, i.e., the ratio of the genetic association with outcome (β_GY_) to the genetic association with exposure (β_GX_) (28) (Supplementary Figure 1, available on the *Arthritis & Rheumatology* website at http://onlinelibrary.wiley.com/doi/10.1002/art.41561/abstract). Wald estimates for multiple variants for the same exposure were combined using inverse variance–weighted (IVW) MR with multiplicative random effects (28), weighted median (29), and MR‐Egger (30) because these methods rely on different assumptions for valid causal inference. The MR‐Egger intercept with *P* < 0.05 indicates the presence of horizontal pleiotropy (30). Directionally consistent results from different methods increase confidence in the results of MR analyses. To orientate the direction of the effects of instruments, we applied Steiger filtering (31). Steiger filtering examines whether the variance explained between each variant–exposure ($R_{\text{Gx}}^{2}$) is larger than the variance explained between each variant–outcome effect ($R_{\text{GY}}^{2}$), and therefore whether the instrument primarily influences the outcome through the exposure (and not vice versa) (31). Two‐sided *P* values are reported throughout, with a Bonferroni correction for multiple testing threshold (*P* ≤ 2 × 10^−3^, given 22 disease traits were considered). Several of the traits examined in this study are likely to share clinical and underlying immunopathogenic features despite their distinct phenotypes; therefore, using this stringent correction provides a balance between reducing false positives and providing rigorous results.

#### Instrument strength

F statistics were calculated for each instrument of GPIbα as $\frac{R^{2}/K}{\left(1‐R^{2}\right)\left(N‐K‐1\right)}$, where R^2^ indicates the proportion of exposure variability explained by the instrument, K indicates the number of instruments, and N indicates the sample size. R^2^ was calculated as 2EAF(1 – EAF)β^2^, where EAF is the effect allele frequency and β is the effect size of the effect allele. Higher F statistic values reflect a lower risk of weak instrument bias (32).

#### Multiple‐trait colocalization analysis

To differentiate whether any putative causal association detected by two‐step MR is driven by a common causal variant across multiple traits (i.e., GPIbα/platelet count/disease) or just confounded by LD, we subsequently performed multiple‐trait colocalization analyses at each locus (7). Under the assumption of a single causal variant within each region, the Bayesian statistical framework quantifies the posterior probability of association (PPA) for each of the possible hypotheses of colocalization (variant sharing) among the 3 traits (all hypotheses are listed in Supplementary Table 2, available on the *Arthritis & Rheumatology* website at http://onlinelibrary.wiley.com/doi/10.1002/art.41561/abstract). A lead variant‐centric approach was applied, where we extracted effect estimates and allele information for all variants within 1 megabase upstream and downstream of the *cis*‐acting protein quantitative trait locus (*cis*‐pQTL) and *trans*‐acting pQTL for each trait (GPIbα/platelet count/disease), respectively. To provide reliable evidence of colocalization, at least 50 variants (with minor allele frequency >1%), including the causal variant of interest, within the test region for all 3 traits were assessed (7). We assigned prior probabilities that a variant is equally associated with 1 trait (p1 = 1 × 10^−4^), 2 traits (p2 = 1 × 10^−6^), and 3 traits (p3 = 1 × 10^−7^), as recommended (7). The PPA for all 3 traits was ≥0.80, which was considered strong evidence of colocalization (7).

MR analyses were performed using the *TwoSampleMR* package, and multiple‐trait colocalization analyses were conducted using the *moloc* package. Results were visualized using the *forestplot* package in the R software platform (version 3.5.1; R Development Core Team).

## RESULTS

#### Genetic instruments for GPIbα and instrument strength

Two conditionally uncorrelated (LD r^2^ < 0.001) pQTLs associated with GPIbα were used as instruments: *cis*‐pQTL (rs72835078 within the *GP1BA* gene) and *trans*‐pQTL (rs11065979 near the *BRAP* gene). The F statistic for *cis*‐pQTL was 48, with 1.4% of the variance in GPIbα explained by *cis*‐pQTL, and the F statistic for *trans*‐pQTL was 50, with 1.5% of the variance in GPIbα explained by *trans*‐pQTL (Supplementary Table 3, available on the *Arthritis & Rheumatology* website at http://onlinelibrary.wiley.com/doi/10.1002/art.41561/abstract).

#### Association of GPIbα with JIA

**Figure 2:**
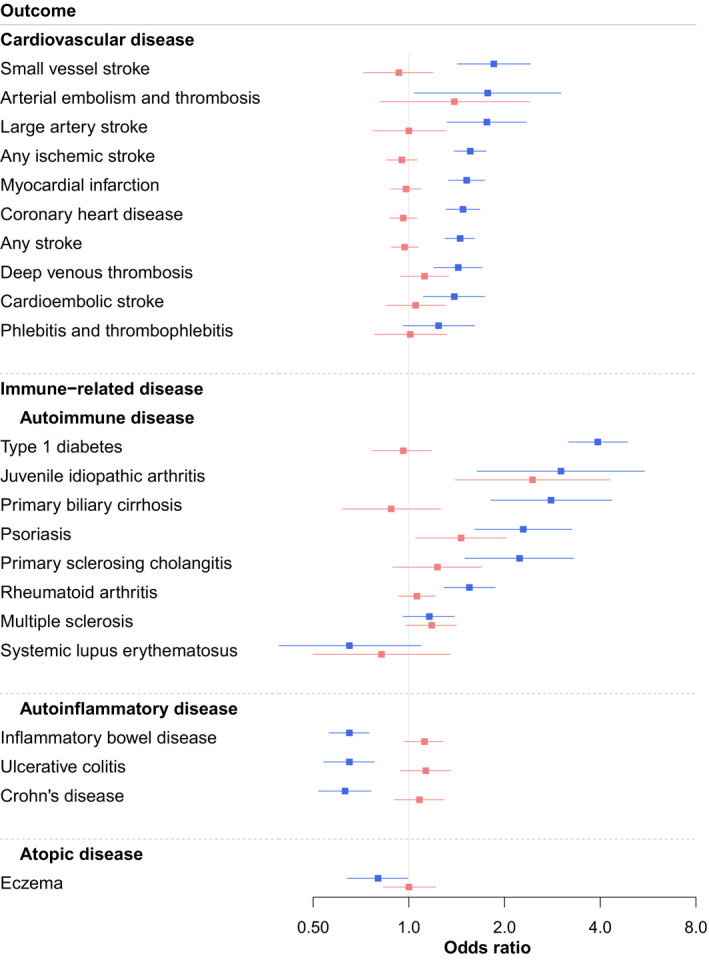
Mendelian randomization estimates for effect of glycoprotein Ib α‐chain on cardiovascular and immune‐related diseases. Values are the odds ratio (point estimate of effect) and 95% confidence interval. Red represents GPIbα instrumented by the *cis*‐acting protein quantitative trait locus (*cis*‐pQTL) within the *GP1BA* gene, and blue represents GPIbα instrumented by the *trans*‐pQTL near the *BRAP* gene.

Using a Bonferroni‐corrected threshold of *P* ≤ 2 × 10^−3^ (equivalent to *P* ≤ 0.05 for a single test), the two‐sample MR analysis (Figure 1A) suggested that increased GPIbα level was positively associated with an increased risk of JIA. Higher GPIbα level instrumented by *cis‐*pQTL was associated with a higher risk of JIA, with an odds ratio (OR) of 2.45 (95% confidence interval [95% CI] 1.40–4.29) (*P* = 1.71 × 10^−3^) (Figure 2 and Supplementary Table 4, available on the *Arthritis & Rheumatology* website at http://onlinelibrary.wiley.com/doi/10.1002/art.41561/abstract). Higher GPIbα level instrumented by *trans‐*pQTL was also associated with a higher risk of JIA (OR 3.01 [95% CI 1.64–5.51], *P* = 3.66 × 10^−4^) (Figure 2 and Supplementary Table 5, available on the *Arthritis & Rheumatology* website at http://onlinelibrary.wiley.com/doi/10.1002/art.41561/abstract). When combining the estimates instrumented by both *cis‐*pQTL and *trans‐*pQTL, per unit increase in GPIbα level was associated with a 169% higher risk of JIA (OR 2.69 [95% CI 1.79–4.06], *P* = 2.33 × 10^−6^).

There was little evidence of an association of GPIbα level instrumented by *cis‐*pQTL with cardiovascular diseases (Figure 2 and Supplementary Table 4). However, the GPIbα *trans‐*pQTL was associated with an increased risk of cardiovascular diseases (small vessel stroke, large artery stroke, any ischemic stroke, MI, CHD, any stroke, and DVT) and autoimmune diseases (type 1 DM, JIA, primary biliary cirrhosis, psoriasis, primary sclerosing cholangitis, and RA) but decreased risk of autoinflammatory diseases (IBD, UC, and CD) (Figure 2 and Supplementary Table 5). The Steiger filtering tests suggested that the instruments primarily influenced the outcome through the exposure (GPIbα).

#### The association of GPIbα with JIA is mediated by platelet count

**Figure 3:**
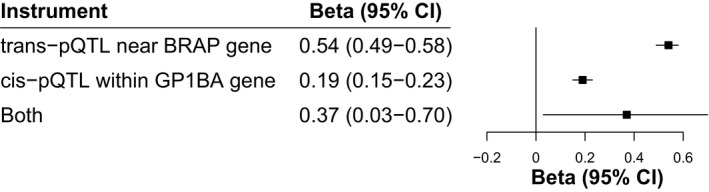
Mendelian randomization estimates for the effect of glycoprotein Ib α‐chain (GPIbα) on platelet count. Values are the beta (point estimate of effect) and 95% confidence interval (95% CI). pQTL = protein quantitative trait locus.

**Figure 4:**
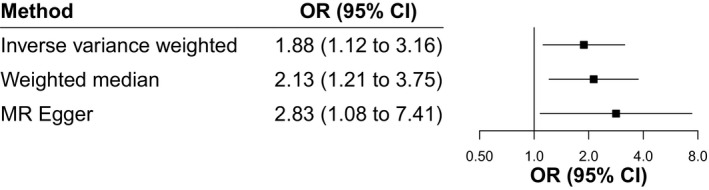
Mendelian randomization (MR) estimates for effect of platelet count on juvenile idiopathic arthritis. Values are the odds ratio (OR; point estimates of effect) and 95% confidence interval (95% CI).

Figure 1B demonstrates how two‐step MR estimates whether the effect of GPIbα on JIA is mediated by platelet count. In the first step, increased GPIbα level was associated with higher platelet count (β = 0.37 [95% CI 0.03–0.70]; *P* = 0.03), among which the effect instrumented by *trans*‐pQTL (β = 0.54 [95% CI 0.49–0.58]; *P* = 1.37 × 10^−143^) was larger than that instrumented by *cis*‐pQTL (β = 0.19 [95% CI 0.15–0.23]; *P* = 2.54 × 10^−19^) (Figure 3). In the second step, there were 135 conditionally uncorrelated variants (LD r^2^ < 0.001) associated with platelet count (*P* < 8.31 × 10^−9^) (Supplementary Table 6, available on the *Arthritis & Rheumatology* website at http://onlinelibrary.wiley.com/doi/10.1002/art.41561/abstract). Using the IVW method, genome‐wide genetically predicted platelet count was positively associated with the risk of JIA (OR 1.88 [95% CI 1.12–3.16], *P* = 0.02) (Figure 4). The weighted median and MR‐Egger methods provided consistent findings, with no evidence of horizontal pleiotropy (Supplementary Table 7, available on the *Arthritis & Rheumatology* website at http://onlinelibrary.wiley.com/doi/10.1002/art.41561/abstract). Sensitivity analyses restricted to each specific instrument for GPIbα also showed that platelet count increased the risk of JIA (Supplementary Table 7).

#### Multiple‐trait colocalization analysis supports the causal association of GPIbα mediated by platelet count with JIA

The two‐step MR analyses suggested that GPIbα mediated by platelet count has an impact on JIA (*P* ≤ 2 × 10^−3^); this association was assessed using multiple‐trait colocalization analysis. The association (GPIbα instrumented by *trans*‐pQTL/platelet count/JIA) was supported by strong evidence of colocalization (PPA ≥ 0.80), indicating that the same causal variant affects 3 traits. The association of GPIbα instrumented by *cis*‐pQTL meditated by platelet count with JIA could not be assessed because the causal variant of interest was not available for the outcome (Supplementary Table 8, available on the *Arthritis & Rheumatology* website at http://onlinelibrary.wiley.com/doi/10.1002/art.41561/abstract).

## DISCUSSION

In this MR study, we observed that GPIbα instrumented by *cis‐*pQTL and GPIbα instrumented by *trans*‐pQTL both increased the risk of oligoarticular and RF‐negative polyarticular JIA. We found no evidence of an association of GPIbα instrumented by *cis‐*pQTL with cardiovascular diseases. However, GPIbα instrumented by *trans*‐pQTL increased the risk of cardiovascular and autoimmune diseases but decreased the risk of autoinflammatory diseases, suggesting potential pleiotropic effects of this *trans*‐pQTL on multiple disease outcomes. Two‐step, two‐sample MR analysis showed that the effect of GPIbα on the increased risk of oligoarticular and RF‐negative polyarticular JIA was mediated by platelet count, which was supported by strong evidence of colocalization. Apart from the well‐established role of platelets in thrombosis (4), our findings provide novel evidence that platelets are causally implicated in oligoarticular and RF‐negative polyarticular JIA.

The GPIb–IX–V complex is a well‐characterized adhesion receptor for vWF and collagen, of which the subunit GPIbα is associated with an increased risk of ischemic cerebrovascular disease in genetic studies (33, 34). Murine data show that absence of GPIbα significantly reduces platelet count and down‐regulates atherosclerosis and inflammation (35), consistent with our findings. Our results also align with a large GWAS of 1 million participants of European ancestry which found that the lead variant at the *BRAP* gene (rs11065979) was positively associated with cardiometabolic and autoimmune diseases in overall and sex‐specific analyses (36). Activated platelets secrete a wide range of cytokines (e.g., interleukin‐6 [IL‐6] and IL‐1), neutrophil chemoattractant (e.g., IL‐8), growth factors, and potent vasoconstrictors (e.g., thromboxane) (4), which play an important role in amplifying inflammatory and thrombotic cascades in these conditions (37, 38). Consistent with our findings, in vivo, platelet‐derived cellular microparticles have been observed in synovial fluid from patients with inflammatory polyarthropathies (e.g., RA, JIA, and psoriatic arthritis) but not from patients with noninflammatory arthritis (osteoarthritis) (37). Furthermore, platelet indices were associated with increased disease activity and severity of JIA (oligoarticular, RF‐negative polyarticular, and systemic subtypes) and were highly labile, particularly in the acute phase (39).

Our findings are consistent with those of a growing number of studies that illustrate the close relationship between atherosclerotic and immune‐mediated disorders (40), leading to the exploration of the role of antiatherosclerotic agents in the autoimmune arena. The antiplatelet agent ticagrelor is under investigation in RA (Clinicaltrials.gov identifier: NCT02874092), and abciximab (a glycoprotein IIb/IIIa inhibitor) is used in children with Kawasaki disease (an inflammatory vasculitis that particularly affects the heart) (41). With regard to the role of platelets in JIA, JIA patients have been shown to have impaired vascular function and thus potentially increased cardiovascular risk (42). Existing therapies for JIA include nonselective nonsteroidal antiinflammatory drugs, which have been shown to antagonize platelet function, and escalation to biologic therapies including anti–tumor necrosis factor (e.g., infliximab, adalimumab, and golimumab), anti–IL‐6 (tocilizumab), and anti–IL‐1 (canakinumab and anakinra) (43). IL‐1 blockade with anakinra has limited efficacy in RA (44), and it has been postulated that this is, in part, due to difficulty in antagonizing platelet microparticle–derived IL‐1 (37). Conversely, IL‐1 blockade is highly effective in the treatment of systemic JIA (45), where very high platelet counts are common.

In our study, GPIbα was associated with an increased risk of both oligoarticular and RF‐negative polyarticular JIA, and this association was shown to be mediated by platelet count. Our findings imply a novel role for platelets in oligoarticular and RF‐negative polyarticular JIA, extending the pathogenic role of platelets in JIA to include disease causation. Therefore, GPIbα represents a potential new therapeutic strategy or a drug repurposing opportunity for these JIA subtypes, which is supported within the current literature. However, given multiple physiologic drivers and functions of platelets (46), such approaches need to be carefully explored to ensure therapeutic benefit. In addition, JIA consists of 7 subtypes (of which oligoarticular and polyarticular subtypes account for up to 90%) (47), and it is increasingly recognized that these comprise discrete clinical entities (48). Further work will be required to ascertain whether these findings are applicable to other JIA subtypes, in particular systemic JIA.

The limitations of this study include, first, that ~8% of the participants from the INTERVAL study (3,300 of 40,521) overlapped between the proteome GWAS and the hematologic GWAS. Nonetheless, bias due to sample overlap is likely to be negligible in this study due to the presence of strong instruments (49). Second, exposures instrumented by a single variant precluded the use of pleiotropy‐robust MR methods, such as weighted median and MR‐Egger (29, 30), which require a large number of instruments. Therefore, to improve the reliability of causal inference, we used multiple‐trait colocalization to complement the MR findings, as recommended (50). Third, it is important to note that although multiple‐trait colocalization analysis provided strong evidence that GPIbα impacts disease via its effects on platelet count, other potential interpretations such as horizontal pleiotropy should be considered. Fourth, *BRAP* also associates with 3 other proteins (vascular cell adhesion molecule 1, β_2_‐microglobulin, and CXCL16), which may also play a role (3). Nevertheless, these proteins are also on the same biologic pathway as GPIbα (9). Fifth, we used platelet count as the mediator; however, platelet count alone may not represent a major or sole determinant of thrombosis and inflammation, and other platelet indices may also be important. Sixth, genetic contributions to complex traits are partitioned into effects from *cis‐*genes and *trans‐*genes (51). However, authoritative analysis conclusively assessing gene regulatory networks on complex traits is beyond the scope of this study. Seventh, summary statistics are subject to the quality control and covariable adjustments conducted by the original researchers of the GWAS based on the specific optimization requirements of their data sets; the use of summary statistics precluded re‐adjustment of data. Finally, this investigation was conducted using summary statistics obtained from participants of European ancestry, and therefore might not be generalizable to other ethnic populations (52). Replication of our findings in other ethnic populations will be helpful to improve the generalizability, and evaluate whether there are underlying ethnic differences in the pathogenesis of disease (53), once data become available.

Using two‐step MR and multiple‐trait colocalization approaches, we provide reliable genetic evidence that the genetic variants that regulate GPIbα proteomic pathways, with well‐characterized biology function on platelet count, have a causal etiologic role in oligoarticular and RF‐negative polyarticular JIA. Our findings highlight the active role of platelets in these JIA subtypes, and GPIbα as a putative therapeutic target for these JIA subtypes.

## AUTHOR CONTRIBUTIONS

All authors were involved in drafting the article or revising it critically for important intellectual content, and all authors approved the final version to be published. Dr. Luo had full access to all of the data in the study and takes responsibility for the integrity of the data and the accuracy of the data analysis.

### Study conception and design

Luo, Clarke, Ramanan, Schooling, Gaunt, Au Yeung, Zheng.

### Acquisition of data

Luo, Thompson, Langefeld, Marion, Grom, Gaunt, Zheng.

### Analysis and interpretation of data

Luo, Clarke, Ramanan, Schooling, Gaunt, Au Yeung, Zheng.

## Supporting information

Fig S1

Table S1‐S8
