# Supplementary figures and images for: Platelet Glycoprotein Ib α‐Chain as a Putative Therapeutic Target for Juvenile Idiopathic Arthritis: A Mendelian Randomization Study
Source: Arthritis Rheumatol. 2021 Feb 21;73(4):693–701. doi: 10.1002/art.41561 (PMC8048917; doi:10.1002/art.41561)

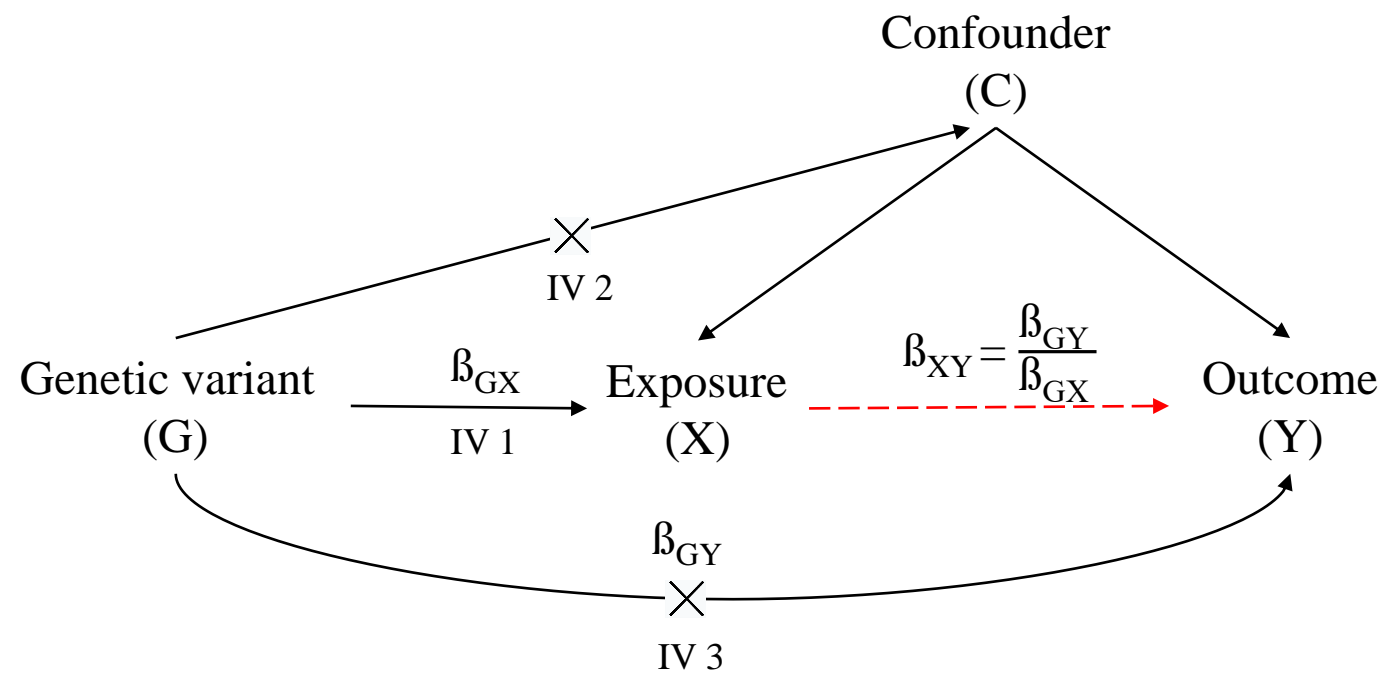

Supplement: Supplementary file 1 — Fig S1 [file ART-73-693-s002.pdf]
